# *Nono* deficiency compromises TET1 chromatin association and impedes neuronal differentiation of mouse embryonic stem cells

**DOI:** 10.1093/nar/gkaa213

**Published:** 2020-04-14

**Authors:** Wenjing Li, Violetta Karwacki-Neisius, Chun Ma, Li Tan, Yang Shi, Feizhen Wu, Yujiang Geno Shi

**Affiliations:** 1 Laboratory of Epigenetics, Institutes of Biomedical Sciences, Fudan University, Shanghai, 200032, China, and Key Laboratory of Birth Defects, Children's Hospital of Fudan University, Shanghai, 201102, China; 2 Endocrinology Division, Brigham and Women's Hospital, Harvard Medical School, 221 Longwood Avenue, Boston, MA 02115, USA; 3 Division of Newborn Medicine and Program in Epigenetics, Boston Children's Hospital, 300 Longwood Avenue, Boston, MA, 02115, USA and Department of Cell Biology, Harvard Medical School, 240 Longwood Avenue, Boston, MA 02115, USA

## Abstract

NONO is a DNA/RNA-binding protein, which plays a critical regulatory role during cell stage transitions of mouse embryonic stem cells (mESCs). However, its function in neuronal lineage commitment and the molecular mechanisms of its action in such processes are largely unknown. Here we report that NONO plays a key role during neuronal differentiation of mESCs. *Nono* deletion impedes neuronal lineage commitment largely due to a failure of up-regulation of specific genes critical for neuronal differentiation. Many of the NONO regulated genes are also DNA demethylase TET1 targeted genes. Importantly, re-introducing wild type NONO to the Nono KO cells, not only restores the normal expression of the majority of NONO/TET1 coregulated genes but also rescues the defective neuronal differentiation of *Nono*-deficient mESCs. Mechanistically, our data shows that NONO directly interacts with TET1 via its DNA binding domain and recruits TET1 to genomic loci to regulate 5-hydroxymethylcytosine levels. *Nono* deletion leads to a significant dissociation of TET1 from chromatin and dysregulation of DNA hydroxymethylation of neuronal genes. Taken together, our findings reveal a key role and an epigenetic mechanism of action of NONO in regulation of TET1-targeted neuronal genes, offering new functional and mechanistic understanding of NONO in stem cell functions, lineage commitment and specification.

## INTRODUCTION

Mouse embryonic stem cell (mESC) pluripotency is facilitated by a gene regulatory network centered around the transcription factors OCT4, SOX2 and NANOG, which control the dual abilities of mESCs to self-renew and to differentiate (1–5). In addition to these established factors we recently identified *Nono* (also known as *Nrb54* and *P54nrb*) as a novel player in the control of mESC pluripotency where NONO acts as a chromatin regulator, cooperating with ERK to regulate the integrity of bivalent domains, which control the balance between self-renewal and differentiation (6).

NONO was originally identified as a non-POU domain-containing, octamer binding protein (7). It binds RNA and DNA, possibly via its helix-turn-helix (HTH) and the RNA recognition motif (RRM) domains (7–10). NONO protein plays important roles in diverse cellular contexts including mRNA splicing (9–15), transcriptional regulation (14–20), double strand DNA break repair (21–24), circadian clock regulation (25), paraspeckle formation (26) and also acts as an innate immune sensor of the HIV capsid in the nucleus (27). Interestingly, not only do *Nono*-deficient mice exhibit cognitive and affective deficits, but mutations in *NONO* are also observed in patients with intellectual disability (28), indicating a critical role of NONO in neurodevelopment. However, the understanding of the molecular mechanisms by which NONO contributes to neuronal lineage specification is incomplete.

Ten-Eleven Translocation 1 (TET1), a founding member of the methylcytosine dioxygenase family, is capable of successively oxidizing 5-methylcytosine (5mC) modifications of DNA to 5-hydroxymethylcytosine (5hmC) (29–31), 5-formylcytosine (5fC) and 5-carboxylcytosine (5caC) (32,33). TET proteins have been implicated in gene expression regulation, cell fate determination, and cancer development (34–43). TET1 is highly expressed in the inner cell mass of the blastocyst, primordial germ cells and mESCs, where it acts as a critical component of the pluripotency regulatory network (38–40,44,45). TET1 has been shown to be enriched at transcriptional start sites of CpG-rich promoters and gene bodies in mESCs, where it promotes DNA demethylation and modulates gene transcription (35–38,46–48). Functionally, *Tet1*-depleted mESCs form teratomas, which not only show emergence of trophoblastic giant cells and increased endodermal differentiation but also reduced neuroectoderm formation (44,45). Interestingly, *Tet1*-deficient mice display behavioral abnormalities and defects in learning, memory, and expression of neuronal activity related genes (49), suggesting an essential role for TET1 in neurodevelopment. However, the molecular mechanisms by which TET1 contributes to these neuronal processes and functions are still largely unknown.

Here, we report that NONO is critical for neuronal lineage commitment of mESCs. Nono KO mESCs fail to upregulate TET1-targeted neuronal genes during neuronal differentiation, a phenotype that could be rescued by restoring wild type NONO in Nono KO cells. Mechanistically, we show that TET1 is an integral component of the NONO complex. NONO directly interacts with TET1 via its DNA binding domain through which NONO recruits TET1 to genomic loci to orchestrate the 5mC to 5hmC transitions, specifically at genes important for neuronal differentiation. We envision that the collaboration between NONO and TET1 at the naïve cell stage is pivotal for the initiation of proper neuronal differentiation dynamics.

## MATERIALS AND METHODS

### Mouse embryonic stem cell cultures

Nono KO and Nono KO + WT mESCs were engineered as described previously (6). All lines were cultured in DMEM containing 10% fetal calf serum (Gibco, cat no. 16000-044), 100 Units/ml leukemia inhibitory factor (Millipore, cat no. ESG1107), 1× MEM non-essential amino acids (Invitrogen cat.no. 11140050) and 100 mM 2-mercaptoethanol (Gibco cat no. 21985-023). Cells were cultured on 0.1% gelatin (Sigma Aldrich, cat no. G1393-100ml) coated tissue culture flasks.

### Monolayer neuronal differentiation procedures

mESCs were plated in a six-well plate at a density of 1 × 10^5^ cells/well in standard mESC medium with LIF (100 units/ml) overnight. On the next day cells were washed with 1xPBS and mESC medium was changed to neural maintenance medium. Cells were cultured for 12 days in neural maintenance medium.

Neural maintenance medium is composed of a 1:1 mixture of N-2 (Gibco, cat no. 17502048) and B-27 (Gibco, cat no. A35828-01)-containing media.

N-2 medium consists of DMEM/F-12 (Fisher scientific, cat no. mt10092cv), 1× N-2, 5 μg ml^−1^ insulin (Sigma-Aldrich, cat no. I9278-5M), 1 mM l-glutamine (Corning, cat no. 25-005-CL), 100 μm nonessential amino acids (Invitrogen, cat no. 11140050), 100 μM 2-mercaptoethanol (Gibco, cat no. 21985-023), 50 U ml^−1^ penicillin and 50 mg ml^−1^ streptomycin (Corning, cat no. 30-002-CL).

B-27 medium consists of Neurobasal (Life Technologies, cat no. 21103-049), 1× B-27, 200 mM l-glutamine, 50 U ml^−1^ penicillin and 50 mg ml^−1^ streptomycin.

### Immunofluorescence analysis

Cells were washed carefully with 1× PBS and fixed with 4% paraformaldehyde for 15 min at room temperature, permeabilized with 0.1% Triton X-100 in PBS for 5 min and blocked with 3% serum, 1% BSA in PBS 0.1% Triton X-100 at RT for 30–60 min. Incubation with primary antibodies diluted in blocking solutions was performed overnight at 4°C. Cells were washed (four washes, each 10 min with 1× PBS) and secondary antibodies were incubated in blocking solution for 1 h. Secondary antibodies were used at 1/1000 dilutions (Alexa Fluor, Thermo Fisher scientific). Cells were counterstained with Hoechst. Primary antibodies used in this study were TUJI (Covance, cat no. MMS-435P) and OCT4 (Santa Cruz Biotechnology, cat no. sc-5279).

### Co-Immunoprecipitation

Freshly made nuclear extracts were purified as previously described (50). Nuclear extracts were lightly sonicated (15 s ON and 45 s OFF), then centrifuged at 12 000 rpm for 10 min at 4°C. Supernatants were incubated with 2 μg of NONO antibody, TET1 antibody and control IgG, respectively, followed by addition of 15 μl of protein A/G agarose beads (Millipore). Incubation was performed at 4°C overnight. Beads were then washed five times with washing buffer (50 mM Tris–HCl pH 7.9, 150 mM KCl, 5 mM MgCl_2_, 0.2 mM EDTA, 5% glycerol, 0.1% NP-40, 3 mM β-ME and protease inhibitors). 50 μl SDS loading buffer was added to washed beads and then boiled for 10 min for Western blot analyses.

### *In vitro* pull-down assay

Recombinant proteins GST, GST-TET1CD and Flag-NONO were purified from Sf9 insect cells. A total of 5 μg Flag-NONO was incubated with 5 μg CST and 5 μg GST-TET1 separately in a 200 μl reaction in binding buffer (50 mM Tris–HCl pH 8.0,150 mM NaCl, 0.1% Triton X-100) for 3 h at 4°C. Incubation with Flag beads for 1 h at 4°C followed. Flag beads were then washed five times with 500 μl of binding buffer. The bound proteins were subjected to Western blot analysis and Commassie Blue staining by SDS/PAGE.

### Immunoblotting

Western blotting was performed as described (51). Briefly, whole cell lysates (100 μg) were resolved on a 8% SDS-PAGE gel, transferred to nitrocellulose membranes and blotted for anti-NONO at a 1:3000 dilution (Santa Cruz Biotechnologies, cat no. sc-166702) and anti-TET1 at a 1:3000 dilution (the TET1 antibody was a kind gift from Dr Guoliang Xu). The secondary antibody, anti-rabbit IgG-peroxidase (Sigma, A6154), was used at a 1:5000 dilution. The peroxidase activity was visualized with the SuperSignal West Pico Kit (Pierce).

### Identification of the NONO protein complex in mESCs

Tandem affinity purification was performed as described (52). To identify potential NONO partners, we performed tandem affinity purification (TAP) for the NONO complex by generating a mESC line stably expressing Flag-HA-Nono, which we then purified with an anti-Flag-HA antibody. MS/MS analysis was used to further verify the components of the complex.

The Flag-HA-Nono knock-in mESC line was constructed by cloning the NONO open reading frame into the pPB Flag-HA expression vector. Nuclear extracts from Flag-HA-Nono knock-in mESCs were prepared as previously described (6). Briefly, forty large culture dishes (15 × 15 cm) were washed with pre-cold PBS containing PMSF. Cells were scrapped and cytoplasmic fraction was removed by incubating cells with buffer A (10 mM HEPES pH 7.6, 1.5 mM MgCl_2_, 10 mM KCl and proteinase inhibitors). Nuclear pellets were then incubated with buffer C (20 mM HEPES pH 7.6, 25% glycerol, 0.42 M NaCl, 1.5 mM MgCl_2_, 0.2 mM EDTA and proteinase inhibitors). Finally, the salt concentration was decreased to 10 mM by dialyzing with buffer D (20 mM HEPES pH 7.6, 20% glycerol, 100 mM KCl, 1.5 mM MgCl_2_, 0.2 mM EDTA) at 4°C for 3 h.

Freshly made nuclear extracts were purified with Flag beads and HA beads separately. After protein purification, protein complexes were boiled and silver staining was performed to visualize proteins by agarose gel electrophoresis on a 10% SDS-PAGE gel. Affinity purification was used to isolate the NONO protein complex for mass spectrometry identification.

### HPLC–MS/MS method

HPLC–MS/MS analysis was performed using the MassHunter System (Agilent). Briefly: extracted genomic DNA was digested via a one-step procedure performed with DNA Degradase™ from Zymo Research, which is a nuclease mix that quickly and efficiently degrades DNA to its individual nucleotide components, for the following whole-genome DNA methylation analysis by HPLC. 1 μg genomic DNA was incubated with 1 μl (10 U) of DNA Degradase™ in a 25 μl reaction volume and was incubated at 37°C for 4 h. The digested samples were then subjected to LC–MS/MS analysis via the MassHunter System manufacturer's protocol. The mass spectrometer was optimized and set up in selected reaction monitoring (SRM) scan mode for monitoring the [M+H^+^] of 5hmC (258.1→142.1), and deoxycytidine (128.1→112.1)

### RNA-seq and data analyses

Total RNA was isolated using the TRIZOL reagent according to the manufacturer's instructions (Life Technologies, cat no. 15596018).

RNA-seq library preparation was carried out according to manufacture's guidelines (Illumina) and our previous protocol (C-10365, Life Technologies (53)).

For RNA-seq data analyses, first the FASTQ data of sequencing reads were trimmed using the program trim_galore (v0.6.4) with parameters,’–paired –illumina’, to remove low quality reads and adapter reads. Consecutively, we mapped the trimmed reads to the mouse genome reference (UCSC mm9) using the software TopHat (v2.1.1) (54) with default parameters. The program Cufflinks (v2.2.1) (54) with default parameters was used to assign the mapped reads to mouse transcripts (UCSC mm9) for identification of the gene expression abundance, represented by FPKM (Fragments Per Kilobase of transcript per Million mapped reads). The FPKM values were normalized to TPM (Transcripts Per Kilobase Million) using the script, FPKM2TPM.R, to allow the comparison of gene expression between samples. The program cuffdiff with default parameters in the Cufflinks suite was used to calculate the fold-change and *P*-value of genes for comparison between our samples. Three biological replicates for day 0 and two biological replicates for day 3, day 6 and day 12 were investigated to identify differentially expressed genes at the cut-off of fold-change >1.5 and *P*-value <0.05. The R program from Bioconductor clusterProfiler (v3.14.0) (55) was used to perform GO term and KEGG pathway enrichment analysis for differential gene expression. The enriched GO terms or KEGG pathways were visualized by using a home-made script, enrichment_plot.R. All R-scripts and middle-data were deposited to our GitHub website (https://github.com/FeizhenWu/Nono).

### ChIP and ChIP-seq and data analyses

As previously described (56), chromatin samples were incubated with specific antibodies in the ChIP Lysis buffer (20 mM Tris–HCl pH 8.1, 300 mM NaCl, 2 mM EDTA, 1% Triton X-100 and 0.05% SDS) overnight at 4°C. The protein-DNA complexes were immobilized on pre-washed protein A/G beads (30 μl per reaction). The bound fractions were washed three times with the lysis buffer, then twice with the low salt wash buffer (10 mM Tris–HCl, 250 mM LiCl, 1 mM EDTA, 0.5% NP-40, 0.5% Na-deoxylcholate), and one time with 10 mM Tris–HCl pH 8.0. De-crosslinking was carried out in the elution buffer (50 mM Tris–HCl pH 8.0, 10 mM EDTA and 1% SDS) at 65°C for 4 h. After 1 h of RNase A and Proteinase K digestion at 55°C, DNA samples were then purified using the PCR extraction kit (QIAGEN #28006).

The precipitated DNA samples were analyzed using real-time quantitative PCR (qPCR) (Roche) and were prepared for deep sequencing according to Illumina's protocol (Illumina and (53)).

For ChIP-seq data analysis, we used the Bowtie2 (v2.3.5) to map the sequencing reads to the mouse genome (UCSC mm9) and identified the significant binding sites (peaks) by using the MACS2 (v2.1.4) with the broad peak mode for NONO sample and with default parameters for other samples (57). The cut-off of peak calling was a *P*-value <1 × 10^−5^. TET1 and NONO ChIP-seq were performed on two biological replicates. To perform the overall correlation analysis between NONO and TET1 binding signals, we used the command: ‘bedtools makewindows -g mm9.genome -w 10000 > mm9.bin10k.bed’, to generate a 10-kb-bin file. The mm9.genome is a file containing chromosome size of the mouse genome (UCSC mm9). We performed the command: ‘bedtools intersect -a mm9.bin10k.bed -b Nono.bed > Nono.10k.bin.counts’, to obtain NONO ChIP-seq reads, and used a similar command to obtain the TET1 ChIP-seq reads. We performed Pearson's correlation of the NONO reads and TET1 reads using the R-script. All R-scripts used to perform these analyses and draw figures were deposited to our GitHub website.

## RESULTS

### *Nono* depletion impairs neuronal differentiation which can be rescued by restoring WT NONO in Nono KO cells

**Figure 1.**
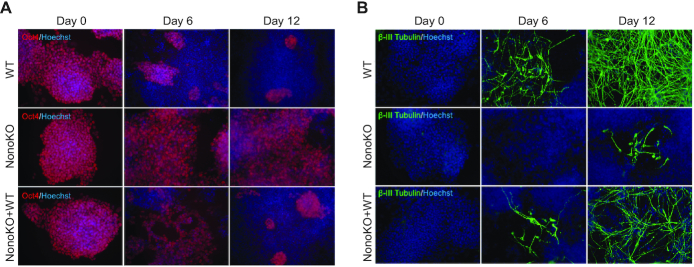
*Nono* knock out impairs neuronal differentiation which can be rescued by restoring WT NONO in Nono KO cells. (**A**, **B**) Immunofluorescence for OCT4 (red) and β-III-TUBULIN (green) at day 0, day 6 and day 12 of neuronal differentiation in WT (E14Tg2a), Nono KO cells, and Nono KO + WT cells. Cells were counterstained with Hoechst (blue).

To understand the functions of NONO in neuronal differentiation, we performed a monolayer neuronal differentiation in WT, Nono KO and Nono KO cells complemented with wild type NONO (Nono KO + WT). Immunofluorescence analysis for the pluripotency factor OCT4 and the neuronal marker β-III-TUBULIN showed that loss of NONO compromises neuronal differentiation as evidenced by much fewer β-III-TUBULIN positive cells at day 6 and 12 and a persistent OCT4 expression throughout the course of differentiation in Nono KO cells (Figure 1A and B). The inappropriate differentiation kinetics could, however, be rescued by the re-expression of NONO in Nono KO + WT cells, though not completely to the extent of what was observed in WT cells (Figure 1A and B).

### Dynamic analysis of gene expression during neuronal differentiation

**Figure 2.**
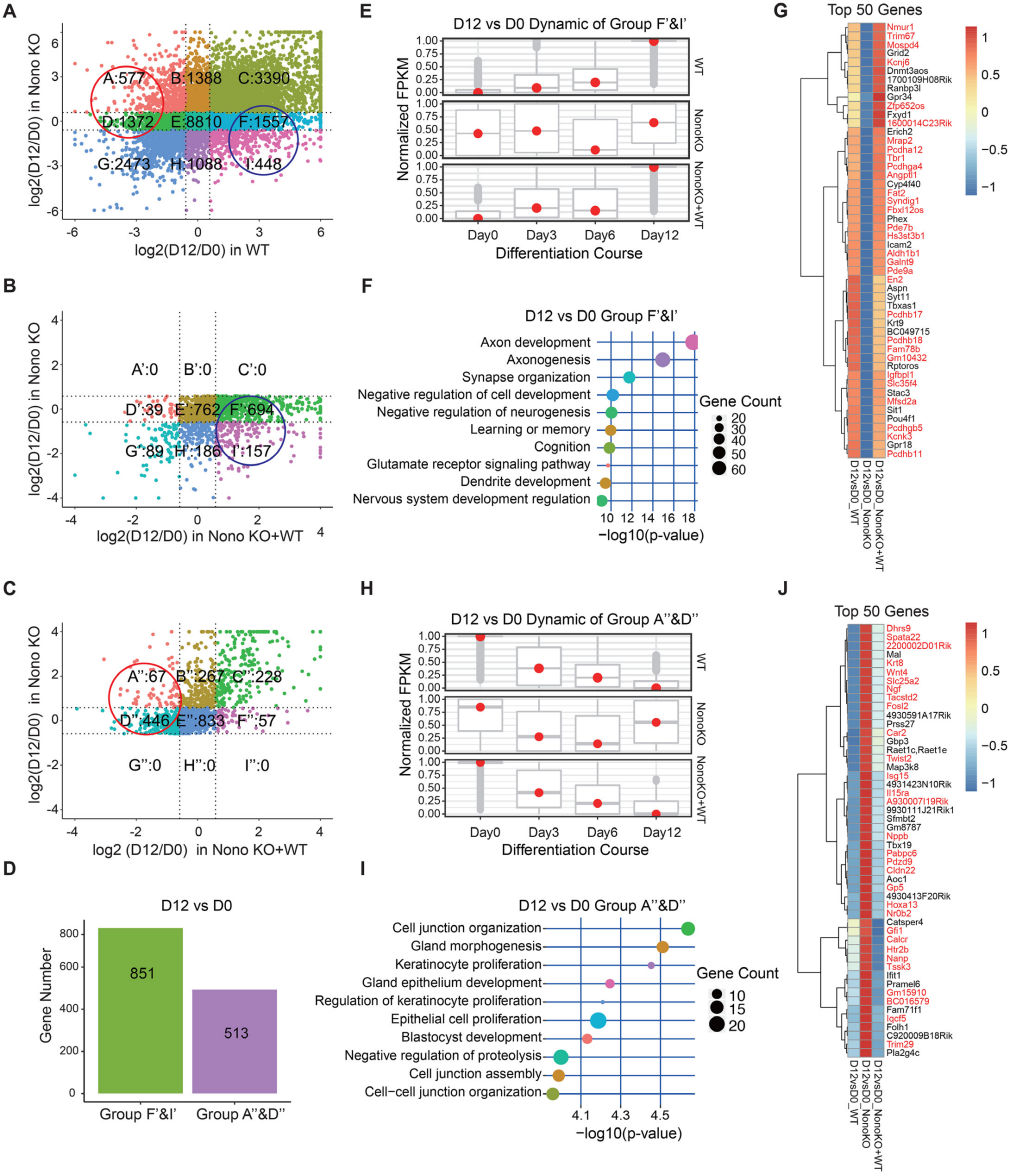
Dynamic analysis of gene expression during neuronal differentiation. (**A**) Comparison of log_2_ gene expression between day 12 and day 0 of neuronal differentiation in WT (E14Tg2a) and Nono KO cells. Genes, that were up- (Group F&I) or down-regulated (Group A&D) in WT cells, were identified. The dot-line in (A), (B) and (C) is a cutoff of log2 (1.5 fold-change). (**B**) Group F&I genes (A) were re-analyzed for the comparison of Nono KO with Nono KO + WT cells. In this step, the number of genes in group F&I (A) was reduced to 851 genes (Group F’&I’). Based on the experimental set-up, these genes depend specifically on NONO expression. (**C**) Group A&D genes (A) were re-analyzed for the comparison of Nono KO with Nono KO + WT cells. In this step, we defined 513 genes (Group A’’&D’’), which are specifically dependent on NONO expression. (**D**) Bar plots illustrate the quantity of genes in group F’&I’ (up-regulated in Nono KO + WT cells) and group A’’&D’’ (down-regulated in Nono KO + WT cells) as analyzed in (B) and (C). (**E**) Box plots show the dynamic expression pattern of group F’&I’ as analyzed in (B) during the complete neuronal differentiation process (day 0 – day 12). The normalized FPKM is represented on the y-axis. The FPKM is equivalent to the average of FPKMs in WT samples, KO samples or Nono KO + WT samples. The FPKM were scaled to values between 0 to 1. (**F**) GO biological process term enrichment analysis for group F’&I’ genes (B). (**G**) Heatmap of the top 50 most differentially expressed genes from group F’&I’ genes (B). Red marked genes represent TET1 target genes. (**H**) Box plots show the dynamic expression pattern of group A’’&D’’ as analyzed in (C) during the complete neuronal differentiation process (day 0 – day 12). The normalized FPKM is represented on the y-axis. The FPKM is equivalent to the average of FPKMs in WT samples, KO samples or Nono KO + WT samples. The FPKM were scaled to values between 0 to 1. (**I**) GO biological process term enrichment analysis for group A’’&D’’ genes (C). (**J**) Heatmap of the top 50 most differentially expressed genes from group A’’&D’’ genes (C). Red marked genes represent TET1 target genes.

To gain insights into the molecular mechanisms underlying *Nono* deletion during neuronal differentiation, we performed a detailed RNA-seq analysis of all three cell lines at days 0, 3, 6 and 12 of neuronal differentiation. In agreement with the cell biology and neuronal phenotype data (Figure 1), the RNA-seq data analyses demonstrated that restoring WT NONO expression in Nono KO cells also largely rescued the molecular phenotype. The comparison of differential expression (DE) of genes in Nono KO relative to WT cells and Nono KO relative to Nono KO + WT cells at days 0, 3, 6 and 12 during neuronal differentiation (Supplementary Figure S1A), demonstrated similar gene expression patterns in WT and Nono KO + WT mESCs (Supplementary Figure S1B). Pearson's correlation analysis between WT and Nono KO + WT cells confirmed these results (Supplementary Figure S1C). To define key genes dependent on NONO expression, we first applied a 9-square plot that classified genes into nine groups according to the log_2_ fold-change at day 12 versus day 0 of neuronal differentiation in both WT (x-axis) and Nono KO cells (y-axis) (Figure 2A). In this first step, we identified genes with increased (Groups F and I, *n* = 2005) and decreased expression in WT cells (Groups A and D, *n* = 1949 (Figure 2A)).

To confirm a true dependency of those genes on NONO expression, we compared their expression in Nono KO (y-axis) versus Nono KO + WT (x-axis) cells (Figure 2B and C). 851 (42%) out of the 2005 genes were up-regulated (Group F’ and I’, Figure 2B and D) and 513 (26%) out of the 1949 genes were down-regulated in Nono KO + WT cells (Group A’’ and D’’, Figure 2C and D). We focused further on these genes, to identify their expression dynamics during neuronal differentiation and key processes impacted by them. As illustrated in Figure 2E by box plots, gene expression progression of Group F’ and I’ over time was identical between WT and Nono KO + WT cells, while expression of these genes remained relatively unaltered in Nono KO cells. GO analysis of these genes identified an enrichment of key neuronal-related pathways such as axon development, learning or memory, synapse organization and nervous system development regulation (Figure 2F). The top 50 most differentially expressed genes from this group included numerous known neuronal regulators including *Nmur1*, *Pcdha12*, *Pcdhb18*, *Tbr1*, *Pde7b* and *Pou4f1* (Figure 2G).

Analysis of the expression dynamics of the genes from Group A’’ and D’’ confirmed similarities between WT and Nono KO + WT cells during neuronal differentiation (Figure 2H). Interestingly, these 513 genes lacked enrichment for neuronal specific GO terms and instead were enriched for GO terms such as cell junction organization, blastocyst development and epithelium cell proliferation (Figure 2I and J). The same analysis of up- and down-regulated genes measured at different time points of neuronal differentiation (day 3/day 0, day 6/day 3 and day 12/day 6) showed similar dynamics and enrichment patterns (Supplementary Figures S2A–D and S3A–F).

In summary, these data not only identified key genes that depend on NONO expression during neuronal differentiation but also suggested that the compromised neuronal differentiation induced by NONO is associated with the altered expression of these critical neuronal regulators.

### *Nono* depletion leads to reduction of TET1 association in the mouse ESC genome

The TET1 protein has been implicated in gene expression regulation and cell fate determination in mESCs (29,37–40). Interestingly, we observed that approximately 50% of the genes identified in Figure 2 were TET1 targets when compared to publicly available gene expression database (Figure 2G and J; marked in red). Given that TET1 has been previously associated with learning and memory impairments in mice (49), and NONO has been shown to be a major component of the TET complex (34), we therefore hypothesized that NONO and TET1 collaborate to enable appropriate neuronal gene regulation.

**Figure 3.**
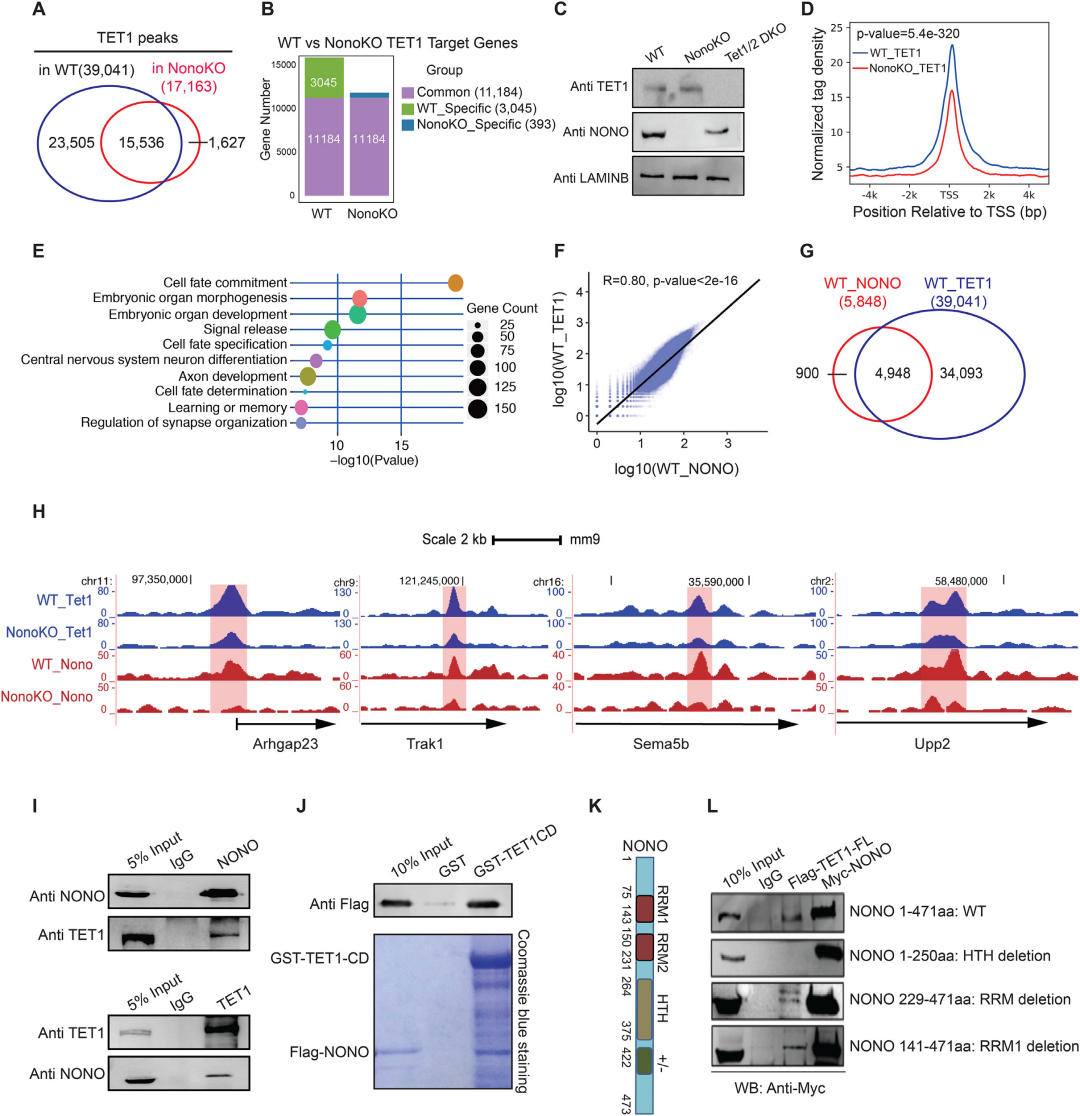
*Nono* deletion leads to reduction of TET1 association in the mouse ESC genome. (**A**) Venn diagram showing overlap of TET1 binding peaks in WT (E14Tg2a) and Nono KO cells. (**B**) Bar plots depicting the quantity of TET1 target genes in WT (E14Tg2a) and Nono KO cells. Gene groups have been divided into ‘Common’, ‘WT-specific’, and ‘Nono KO-specific’ target genes. (**C**) Western blot analysis for NONO and TET1 expression in WT (E14Tg2a), Nono KO and Tet1/2 DKO cell lines. Antibodies are indicated on the left. (**D**) TET1 binding distribution in WT (E14Tg2a) and Nono KO cells at promoter regions of TET1 target genes which have a significantly reduced TET1 binding in Nono KO cells. (**E**) GO biological process term enrichment analysis for genes with reduction of TET1 binding in Nono KO cells. (**F**) Correlation analysis of NONO and TET1 binding signals in WT (E14Tg2a) cells. Pearson's correlation of NONO and TET1 binding signals in nonoverlapping 10-kb bins spanning the mouse genome (mm9) was performed. (**G**) Venn diagram depicting the overlap of NONO and TET1 binding peaks in WT (E14Tg2a) cells. (**H**) UCSC screenshots depicting TET1 and NONO ChIP-seq signals in WT (E14Tg2a) cells and TET1 ChIP-seq signals in Nono KO cell lines at the indicated genes. (**I**) Immunoprecipitation analysis with IgG and NONO antibodies, followed by Western blot analysis using NONO and TET1 antibodies (top panel). Immunoprecipitation with IgG and TET1 antibodies, followed by Western blot analysis using NONO and TET1 antibodies (bottom panel). (**J**) Recombinant GST, GST-tagged TET1 catalytic domain (CD), and Flag-tagged NONO isolated from Sf9 insect cells interact with each other in vitro. Top: Western blot analysis using an Anti-Flag antibody. Bottom: Coomassie blue staining of the SDS/PGE gel. (**K**) Schematic representation of structural elements of the NONO protein. (**L**) Co-Immunoprecipitation analysis showing the indicated mutations in the NONO protein. Western blot analysis showing that the first C-terminal half of NONO, that encompasses the HTH domain, is necessary for the physical interaction between NONO and TET1.

To test this hypothesis, we examined the functional interaction between TET1 and NONO in the naïve mESC state that could affect differentiation processes, by performing ChIP-seq analysis for TET1 in WT and Nono KO cells. We identified 39 041 TET1 binding events in WT cells and 17 163 TET1 binding events in Nono KO cells (Figure 3A), which were enriched at promoter regions (Supplementary Figures S4A and D). Analysis of the genomic distributions of TET1 ChIP-signals around the transcription start sites (TSSs) and across gene bodies showed enrichment at TSSs (Supplementary Figure S4B–C, E–F) consistent with previously reported studies (36). Surprisingly, more than half of the TET1 binding events (23 505 peaks) were significantly reduced upon *Nono* deletion in WT cells (Figure 3A). We refer to these 23 505 peaks as ‘Reduced Tet 1 peaks’ throughout this paper. To identify potential regulatory targets, we analyzed the genes that are associated with the identified TET1 binding peaks at promoter regions. We identified 11 184 common target genes in both WT and Nono KO cells and 3045 target genes that were WT-specific, indicating that one-fourth of TET1 peak associated genes were lost in Nono KO cells (Figure 3B). The reduction of TET1 genomic associations was independent of changes in TET1 protein expression, as both WT and Nono KO cells expressed comparable levels of the TET1 protein (Figure 3C).

Genomic distribution of TET1 binding in WT and Nono KO cells at promoter regions of the 3045 WT cells specific TET1 target genes (Figure 3B) showed that they have a significantly reduced TET1 binding in Nono KO cells (Figure 3D). These results were further confirmed by ChIP-q-PCR using Tet1/2 DKO cells as a negative control (Supplementary Figure S4G). Interestingly, in contrast to the reduction of TET1 binding in Nono KO cells, the NONO binding in Tet1/2 DKO cells at the five analyzed genomic loci was not affected (Supplementary Figure S4G).

To further explore the biological processes affected by loss of NONO in the group of genes that also have reduced TET1 binding, we performed a gene ontology (GO) analysis (Figure 3E). These results identified that NONO-regulated TET1 target genes in the naïve ESC state are associated with processes such as cell fate commitment, embryonic organ development, and were highly enriched in neuronal GO terms, such as central nervous system neuron differentiation, axon development, learning or memory and regulation of synapse organization.

To address the correlation between NONO and TET1, we performed NONO ChIP-seq analysis in WT mESCs and found that the TET1 signal is significantly correlated with NONO on a genome-wide level (Figure 3F) and that the strength of the association increases in parallel to the window size (Supplementary Figure S4H). This analysis identified a total of 5848 NONO peaks (Figure 3G). Significantly 4948 of these peaks (85%) were co-enriched with TET1 on chromatin (Figure 3G and H). Importantly, analysis of the ‘Reduced TET1 Peaks’ in Nono KO cells and the 4948 TET1 and NONO common peaks defined that one-third (1490 of 4948) of these peaks overlap. Taken together, these results suggest that NONO loss compromises TET1 chromatin association.

### Biochemical characterization of the direct interaction between NONO and TET1 in mESCs

We next biochemically purified the NONO complex using the tandem affinity purification (TAP) method (52). MS/MS proteomic analysis identified the core components of the NONO complex, including OGT, SFPQ, NONO and PSPC1 (Supplementary Figure S5A and B). Importantly, the MS/MS proteomic analysis also revealed that TET1 is associated with the NONO complex (Supplementary Figure S5A and B). Western blot analysis further confirmed the results of the MS/MS proteomic analysis (Supplementary Figure S5C). We validated the interaction between NONO and TET1 by co-immunoprecipitation (Co-IP) and reciprocal Co-IP, followed by Western blot detection (Figure 3I). To further determine if NONO interacts directly with TET1, we conducted *in vitro* pull-down assays using recombinant purified proteins to further characterize the interaction. Recombinant GST, GST-tagged TET1 catalytic domain (CD), and Flag-tagged NONO isolated from Sf9 insect cells interacted with each other *in vitro* (Figure 3J). These results showed that TET1 directly interacts with NONO via the TET1 catalytic domain. Importantly, the C-terminal half of the NONO protein that encompasses the HTH domain, is necessary for its interaction with TET1 (Figure 3K and L). These data indicate that NONO recruits TET1 to promoters via physical interactions.

### *Nono* deletion leads to a genome-wide reduction of 5hmC levels

**Figure 4.**
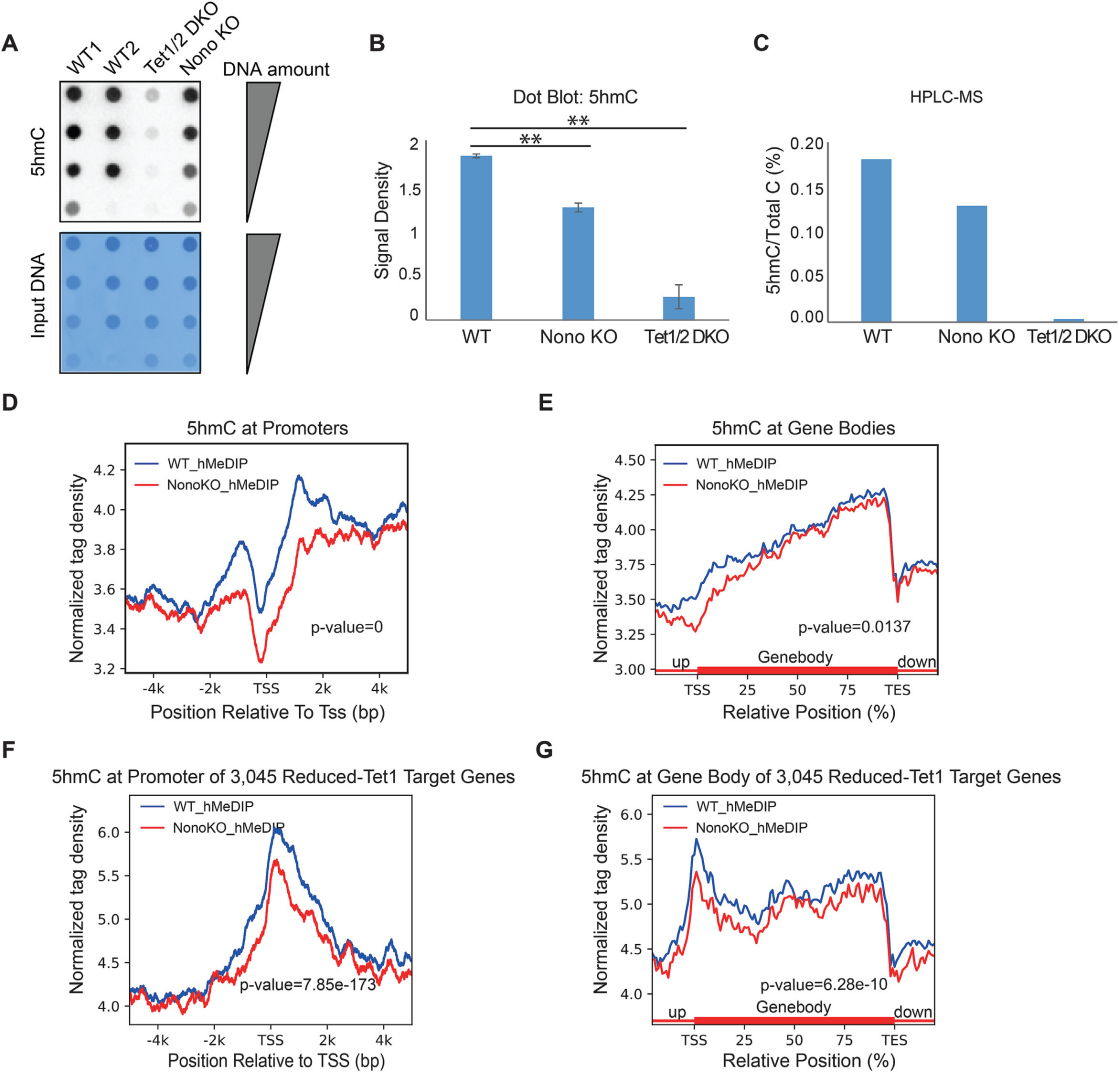
*Nono* deletion leads to a genome-wide reduction of 5hmC levels. (**A**) 5hmC levels in WT (E14Tg2a), Nono KO and Tet1/2 DKO cells measured by dot plot. Methylene staining is shown at the bottom. (**B**) ImageJ quantifications of global 5hmC levels in WT (E14Tg2a), Nono KO and Tet1/2 DKO cells measured by dot blot (A). Error bars represent standard deviation: *n* = 3 biological replicates. For statistical significance we performed an independent two-sample t-test and calculated confidence interval with R Statistical Software (**P< 0.05). (**C**) High performance liquid chromatography (HPLC) of 5hmC signal in WT (E14Tg2a), Nono KO, and Tet1/2 DKO. (**D**, **E**) Genome-wide analysis of 5hmC distribution at gene promoters (D) and gene bodies (E). Refseq genes (mm9) were used as reference annotation for this analysis. (**F**, **G**) Signal plot of the 5hmC distribution at promoter (F) and gene body (G) regions of TET1 target genes that were significantly reduced in Nono KO cells.

Next, we examined if the dissociation of TET1 from chromatin following *Nono* deletion would affect 5hmC levels as TET1 is a well characterized 5mC hydroxylase (30,32,33). We first performed dot blot analysis and found a significant global reduction of 5hmC in Nono KO cells (Figure 4A and B). HPLC analysis of 5hmC levels further confirmed the dot blot results, showing a 25% reduction of 5hmC in Nono KO cells (Figure 4C). To gain insights into TET1-mediated regulation of 5hmC levels at gene specific sites, we performed a genome-wide mapping of 5hmC levels in Nono KO cells using hydroxymethylated DNA immunoprecipitation (hMeDIP). We identified a total of 113 408 5hmC peaks in WT cells and only 76 818 5hmC peaks in Nono KO cells (∼42% of the total 5hmC peak reduction), consistent with dot blot and HPLC results. An analysis of the 5hmC peak distribution in WT and Nono KO cells revealed a significant reduction of 5hmC levels in both gene promoters and gene bodies (Figure 4D and E). In particular, this analysis revealed that 5hmC distribution and level at promoter regions and gene bodies of the TET1 target genes, which have a significantly reduced TET1 binding in Nono KO cells (Figure 3A and B), were significantly reduced (Figure 4F and G).

Additionally, we analyzed the 5mC levels by dot blot analysis, HPLC and MeDIP-seq (Supplementary Figure S6A–D). In contrast to the significant reduction of 5hmC, the levels of 5mC were only slightly affected (Supplementary Figure S6A–D). This is not unexpected as 5hmC is not as abundant as 5mC in the genome. Even Tet1 deletion in mESCs, which has a significant impact on 5hmC levels, only shows a slight reduction of 5mC genome wide (29,36,46). Taken together, these findings suggest that NONO plays an important role in influencing 5hmC levels in mouse ESCs through recruitment of TET1 to genomic sites.

### NONO and TET1 control transcription

**Figure 5.**
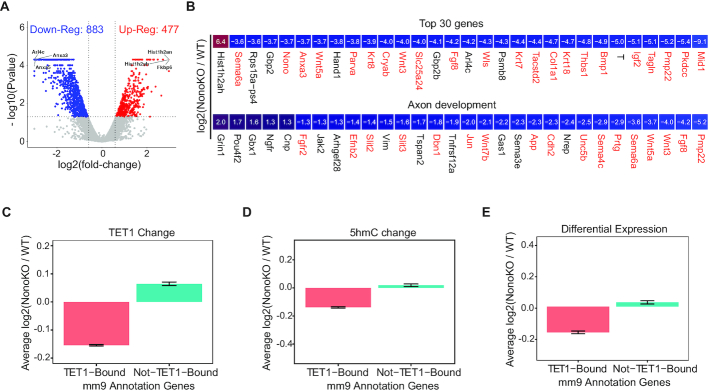
*Nono* deletion leads to downregulation of neuronal differentiation related genes. (**A**) Volcano-plot shows genes differentially expressed in Nono KO relative to WT (E14Tg2a) cells. (**B**) Heatmap shows the log_2_ fold-change of the top 30 most differentially expressed genes in Nono KO relative to WT (E14Tg2a) cells (top-panel). Differentially expressed genes in Nono KO relative to WT cells are related to axon development (bottom panel). Red marked genes show a reduced Tet1 binding at their promoter regions. (**C–E**) Average log_2_ (NonoKO/WT) of Tet1 binding signal (C), 5hmC signal (D) and gene expression (E), of ‘TET1-Bound’ and ‘Not-TET1-Bound’ genes. ‘TET1-Bound’ genes are defined as genes that show TET1 binding at their promoter regions. Refseq genes (mm9) were used as reference annotation for this analysis.

To determine to what extent the dissociation of TET1 from chromatin and the reduction of 5hmC levels in Nono KO cells affects transcription, we re-explored our RNA-seq analysis at the naïve cells stage. We identified 883 down-regulated and 477 up-regulated genes in Nono KO cells (Figure 5A). Importantly, two-thirds of the differentially expressed genes also showed a reduction of TET1 binding in the Nono KO cell line, and the differential expression of genes was statistically associated with TET1 binding level decrease (*P* < 2.2e–16, chi-squared test) (Supplementary Figure S7A). Analysis of the top 30 most differentially expressed genes in Nono KO cells revealed that 29 of the 30 genes were down-regulated and 22 had a reduction of TET1 binding at their promoters (Figure 5B; top and red gene names). Significantly, many of these genes were key developmental genes such as *Bmp1*, *Fgf8*, *Igf2*, *Wnt3a*, *Cryab*, *Anxa3* and *Mid1*. GO analysis of the most differentially expressed genes revealed that they were significantly enriched for terms associated with neuronal processes such as axon development (Figure 5B; bottom and Supplementary Figure S7B).

To obtain further insight into the relationships between gene expression, TET1 binding, and 5hmC levels after NONO loss, we divided the mouse genome reference annotation (mm9) genes into two groups, ‘TET1-bound’ and ‘Not-TET1-bound’. We found that the average TET1 binding level of the ‘TET1-bound’ genes was significantly reduced in Nono KO cells. Intriguingly, ‘Not-TET1-bound’ genes were slightly increased, likely due to the mis-targeting of TET1 to these ‘Not-TET1-bound’ genes after *Nono* deletion (Figure 5C). This result was consistent with the changes of 5hmC distribution (Figure 5D). In agreement with the profile of TET1 binding and 5hmC levels, we observed that ‘TET1-bound’ genes were significantly down-regulated whereas ‘Not-TET1-bound’ genes were only slightly up-regulated, if any, in Nono KO cells (Figure 5E). Collectively, these analyses suggest that NONO and TET1 collaborate to control transcription, which likely involves regulation of 5hmC at NONO/TET1 targeted genes.

## DISCUSSION

Embryonic stem cells have the capacity to self-renew or to differentiate into lineages of all three germ layers (1,2). Based on their outstanding abilities, embryonic stem cells hold a great promise for future stem cell-based therapy and drug development. The investigation of the interplay of epigenetics and gene expression in control of self-renewal and differentiation can therefore provide important insights into fundamental mechanisms how ESCs are regulated (3–5).

In this study, we identified a novel role for NONO in the control of neuronal gene expression regulation. We identified key genes and pathways, which require NONO protein expression for their proper up-regulation during neuronal differentiation. Surprisingly, we found that 50% of these genes were TET1 target genes, suggesting a significant role for TET1 in NONO-regulated gene expression programs. Mechanistically, we identified that the C-terminal half of the NONO protein that encompasses the HTH domain, a major structural DNA binding motif (7–9), is necessary for its interaction with TET1. Our data therefore suggests that NONO recruits TET1 to genomic loci to regulate 5hmC levels. Consistently, NONO loss leads to a drastic dissociation of TET1 from chromatin and dysregulation of DNA methylation and expression of target genes. Out of 39 041 TET1 binding peaks in WT cells, 23 505 peaks were significantly reduced in Nono KO cells. Accordingly, the reduction of 5hmC levels was also observed genome-wide with a predominance at promoter regions. These findings support our hypothesis that NONO regulates TET1 chromatin association to affect gene expression.

Interestingly, GO analysis of genes associated with TET1 and 5hmC level reductions revealed a substantial preference for the neuronal lineage already at the naïve cell stage, suggesting that NONO recruits TET1 preferentially to genes associated with neuronal development prior to differentiation initiation. Out of the top 30 differentially expressed genes in Nono KO cells, the majority were key developmental genes such as *Bmp1*, *Fgf8*, *Igf2*, *Wls* and *Wnt3a*. In addition, other genes important for neuronal development such as *Cryab*, *Anxa3*, *Mid1* and *Pmp22* were highly down-regulated in Nono KO cells. As all these genes had a reduction in TET1 binding and a reduction in the 5hmC levels, we envision that NONO and TET1 collaborate to control transcription through regulation of the 5hmC distribution at promoters of key neuronal genes.

NONO has been reported to cooperate with ERK to control mESC pluripotency. *Nono* knock out diminishes ERK activation and RNA polymerase poising at its target bivalent genes (6). However, a direct role of NONO in the transcriptional regulation involving the recruitment of TET1 and significant alteration of 5hmC has not been reported.

TET1 is essential for DNA demethylation in mESCs, a key epigenetic determinant for cell fate specification (37,39,44,45). Generally, TET protein activity and recruitment are regulated on multiple levels to dictate the final effect on DNA methylation (29,30,36–38,44,47,58). Therefore our finding, that a single factor, NONO, can significantly impact on TET1 association in the genome, is unprecedented.

In addition to the NONO-dependent mechanisms described here, there are likely other uncharacterized mechanisms that regulate TET1 recruitment to genes, which represent promising avenues for future investigations. For example, further studies of the methylome dynamics associated with the expression of various methyltransferases and demethylases during mESC neuronal differentiation are necessary.

Taken together, our study establishes a new molecular mechanism targeting TET1 to specific genomic loci for epigenetic regulation of development and neuronal-related genes and provides a novel connection of the NONO protein to dynamic regulation of the DNA hydroxymethylome which is likely important for mESC functions and lineage specification.

## DATA AVAILABILITY

The RNA- and ChIP-Seq data have been deposited to the NCBI Sequence Read Archive (SRA) database under the accession code: PRJNA527295. All statistical analyses codes, which include all the R-scripts used to perform data analysis as well as to draw figures from the RNA-seq and ChIP-seq data are deposited to the GitHub repository website (https://github.com/FeizhenWu/Nono). All other data sets generated or analyzed during the current study are available from the corresponding author on reasonable request.

## Supplementary Material

gkaa213_Supplemental_File
