# Supplementary material for: Nono deficiency compromises TET1 chromatin association and impedes neuronal differentiation of mouse embryonic stem cells
Source: Nucleic Acids Res. 2020 Apr 14;48(9):4827–38. doi: 10.1093/nar/gkaa213 (PMC7229820; doi:10.1093/nar/gkaa213)

Supplementary legends and figures

Nono deficiency compromises TET1 chromatin association and impedes neuronal differentiation of mouse embryonic stem cells

Wenjing Li[1,2,†], Violetta Karwacki-Neisius[3,†,*], Chun Ma[1], Li Tan[1], Yang Shi[3], Feizhen Wu[1,*], Yujiang Geno Shi[2,*]

[1] Laboratory of Epigenetics, Institutes of Biomedical Sciences, Fudan University, Shanghai, 200032, China, and Key Laboratory of Birth Defects, Children's Hospital of Fudan University, Shanghai, 201102, China.

[2] Endocrinology Division, Brigham and Women's Hospital, Harvard Medical School, 221 Longwood Avenue, Boston, MA, 02115, USA

[3] Division of Newborn Medicine and Program in Epigenetics, Boston Children's Hospital, 300 Longwood Avenue, Boston, MA, 02115, USA and Department of Cell Biology, Harvard Medical School, 240 Longwood Avenue, Boston, MA, 02115, USA

† The authors wish it to be known that, in their opinion, the first 2 authors should be regarded as joint First Authors. These authors contributed equally to this work.

* Correspondence should be addressed to Yujiang Geno Shi. Tel: 1-(617) 525-8097; Fax: (617) 582-6193; Email: yujiang_shi@hms.harvard.edu

* Correspondence may also be addressed to Feizhen Wu (wufz@fudan.edu.cn) and Violetta Karwacki-Neisius (violetta.karwacki-neisius@childrens.harvard.edu).

Supplementary Figure 1. Correlation analysis of gene expression patterns between WT and Nono KO + WT cells

(A) Comparison of differentially expressed genes (DEGs) between two biological replicates (Set 1 and Set 2). The corresponding p-values and Odds Ratios are indicated.

(B) Heatmaps represent a comparison of differential gene expression changes (fold change > 1.5) in Nono KO relative to WT cells (left column) and Nono KO relative to Nono KO + WT cells (right column) at day 0, 3, 6, and 12 of neuronal differentiation. The coefficients were calculated by Pearson correlation. Log2 (fold change) values are color-coded according to the legend on the right.

(C) Scatterplots showing gene expression in Nono KO + WT cells (y-axis) in comparison to WT cells (x-axis) at day 0, 3, 6 and 12 of neuronal differentiation in two biological replicates (Set 1 and Set 2). TPM (transcripts per million) representing gene expression level. Pearson correlation coefficients are represented on the top.

Supplementary Figure 2. Identification of "rescued genes" in Nono KO + WT cells during neuronal differentiation

(A) Comparison of log2 gene expression between day 3 and day 0 (left), day 6 and day 3 (middle), and day 12 and day 6 (right) during neuronal differentiation in WT (E14Tg2a) and Nono KO cells. Genes that were up- (Group F&I) or down-regulated (Group A&D) in WT cells (E14Tg2a) and not in Nono KO cells, were identified. The dot-line in (A), (B), and (C) is the cutoff of log2 (1.5 fold-change).

(B) Group F&I genes (A) were reanalyzed for the comparison of Nono KO with Nono KO + WT cells. Genes in the group F'&I' depend on NONO expression.

(C) Group A&D genes (A) were reanalyzed for the comparison of Nono KO with Nono KO + WT cells. Genes in the group A''&D'' depend on NONO expression.

(D) Bar plots represents the number of gene in Group F'&I' and Group A''&D'' as analyzed in (B) and (C).

Supplementary Figure 3. Dynamic analysis of genes that fail to up- or down-regulate in Nono KO cells during neuronal differentiation

(A-C) Analysis of the stage specific expression patterns of significantly up-regulated genes in Nono KO + WT cells during neuronal differentiation. Stage specific gene expression was determined by comparison of gene expression between day 3 and day 0 (A), day 6 and day 3 (B), and day 12 and day 6 (C). Group F'&I' was identified in Supplementary Figure S2B.

(D-F) Analysis of the stage specific expression patterns of genes that need to be silenced at specific stages of neuronal differentiation but which show a maintained expression in Nono KO cells. Stage specific gene expression was determined by gene expression differences between day 3 and day 0 (D), day 6 and day 3 (E), and day 12 and day 6 (F). Group A''&D'' was identified in Supplementary Figure S2C.

Top left panel: Box plots showing dynamic expression throughout the complete neuronal differentiation process (day 0-day 12) in the indicated group of genes at the indicated time; Bottom left panel: GO enrichment analysis in

the indicated group of genes at the indicated time. Right panel: Heatmap analysis shows the log2 FPKM of the top 50 most differentially expressed genes in WT cells at the indicated time. Scaled FPKM values are color-coded according to the legend on the right. TET1 target genes are marked in red.

Supplementary Figure 4. Genome-wide distribution of TET1 and identification of the NONO complex.

(A) Genomic distribution of TET1-binding events in WT (E14Tg2a) cells.

(B-C) TET1 normalized density distribution at promoters (B) and gene bodies (C) in WT (E14Tg2a) cells.

(D) Genomic distribution of TET1-binding events in Nono KO cells.

(E-F) TET1 normalized density distribution at promoters (E) and gene bodies (F) in Nono KO cells. Refseq genes (mm9) was used as reference annotation for this analysis.

(G) TET1 and NONO ChIP-qPCR analyses at 5 independent gene loci in WT (E14Tg2a), Nono KO, and Tet1/2 DKO cells.

(H) Scatterplots showing the similarities of the TET1 and NONO protein distribution in the genome using different scales (1 kb, 3 kb, 5 kb, and 10 kb). Pearson correlation coefficients are represented on the top.

Supplementary Figure 5. Identification of the NONO complex.

(A) Tandem affinity purification of the NONO complex. Complex components: TET1, OGT, SFPQ, NONO, PSPC1 are indicated.

(B) Unique peptide numbers in the NONO complex as identified by mass spectrometry.

(C) Western blot analysis showing that NONO and TET1 co-purify in the NONO complex. Antibodies are indicated on the left.

Supplementary Figure 6. Genome-wide analysis of 5mC levels

(A) 5mC levels in WT (E14Tg2a), Nono KO, and Tet1/2 DKO cells measured by dot plot. The bottom panel depicts methylene staining.

(B) High performance liquid chromatography (HPLC) of 5mC signal in WT (E14Tg2a), Nono KO, and Tet1/2 DKO.

(C-D) Genome-wide analysis of 5mC distribution at gene promoters (C) and gene bodies (D) in Nono KO cells. Refseq genes (mm9) was used as reference annotation for this analysis.

Supplementary Figure 7. Nono KO leads to a genome-wide reduction of TET1 binding and leads to a down-regulation of neuronal differentiation related genes.

(A) Four square table shows the number of differentially expressed genes which also have a reduction of TET1 binding. Chi-square test shows that there exists a statistical correlation between TET1 binding and differential gene expression. Fold change >1.5 and p-value <0.05 were used as cutoff value for the differentially expressed genes.

(B) GO enrichment analysis for down-regulated genes in Nono KO relative to WT (E14Tg2a) cells.

Supplementary Figure 1

A

Comparison	Set1 DEGs*	Set2 DEGs*	Overlap DEGs**	p-value	Odds Ratio
D0_NonoKO/D0_WT	2802	4347	1784	0e+00	12.7
D3_NonoKO/D3_WT	3911	3501	2393	0e+00	26.9
D6_NonoKO/D6_WT	5807	5577	3621	0e+00	13.7
D12_NonoKO/D12_WT	6374	5741	3532	0e+00	8.6
D0_NonoKO/D0_RE	1009	788	299	1.8e-213	19.3
D3_NonoKO/D3_RE	1538	770	332	2.6e-195	13.8
D6_NonoKO/D6_RE	2397	1553	762	0e+00	12.2
D12_NonoKO/D12_RE	3572	2066	1217	0e+00	11.9

B

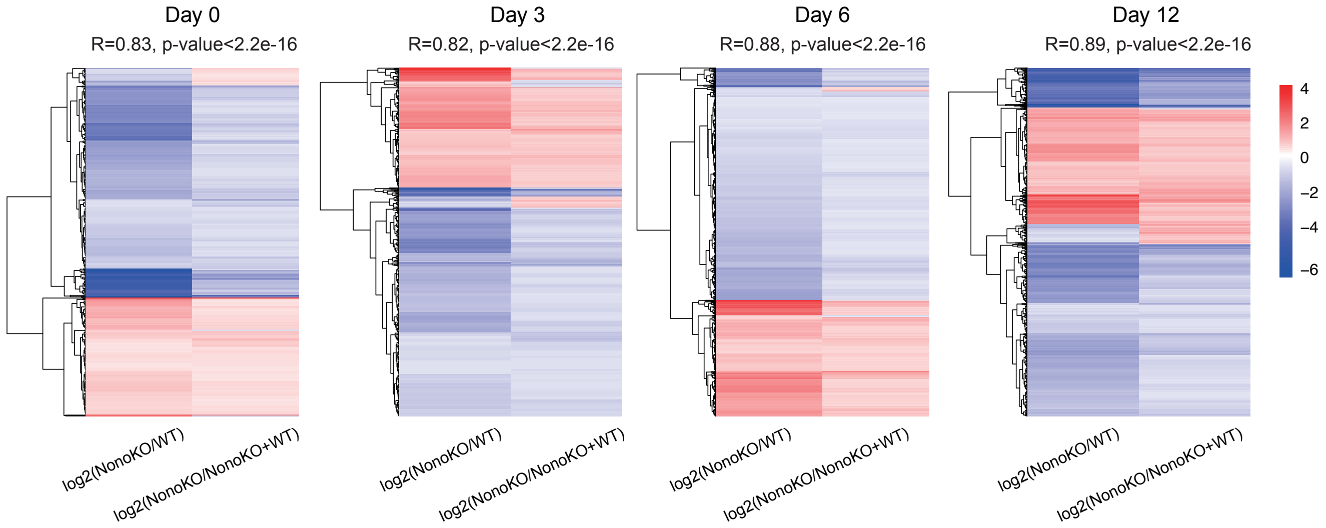

C

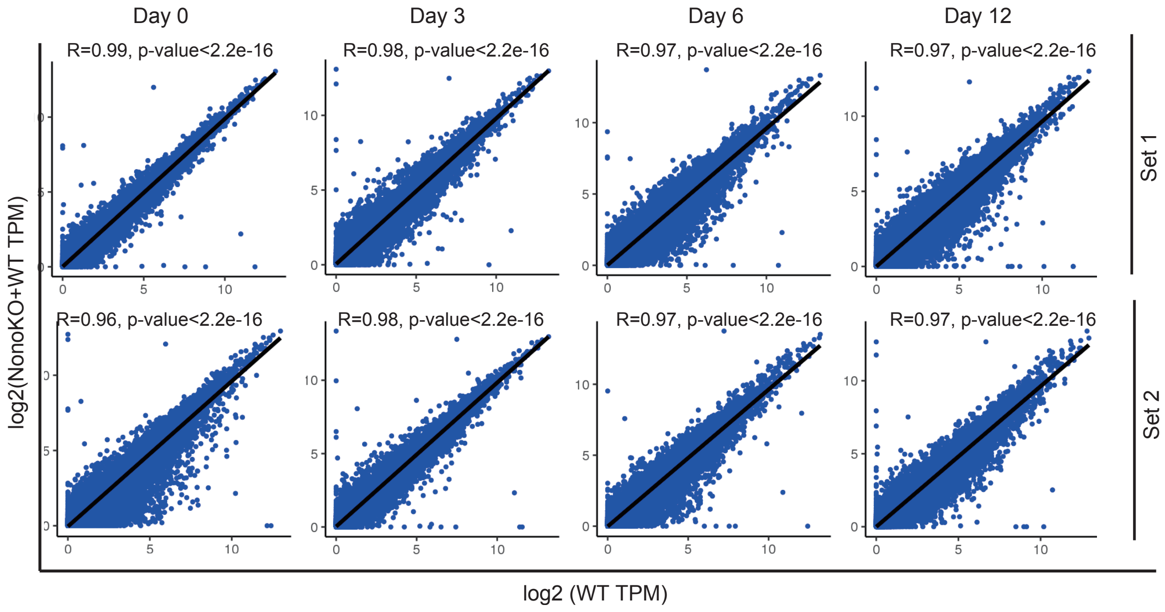

Supplementary Figure 2

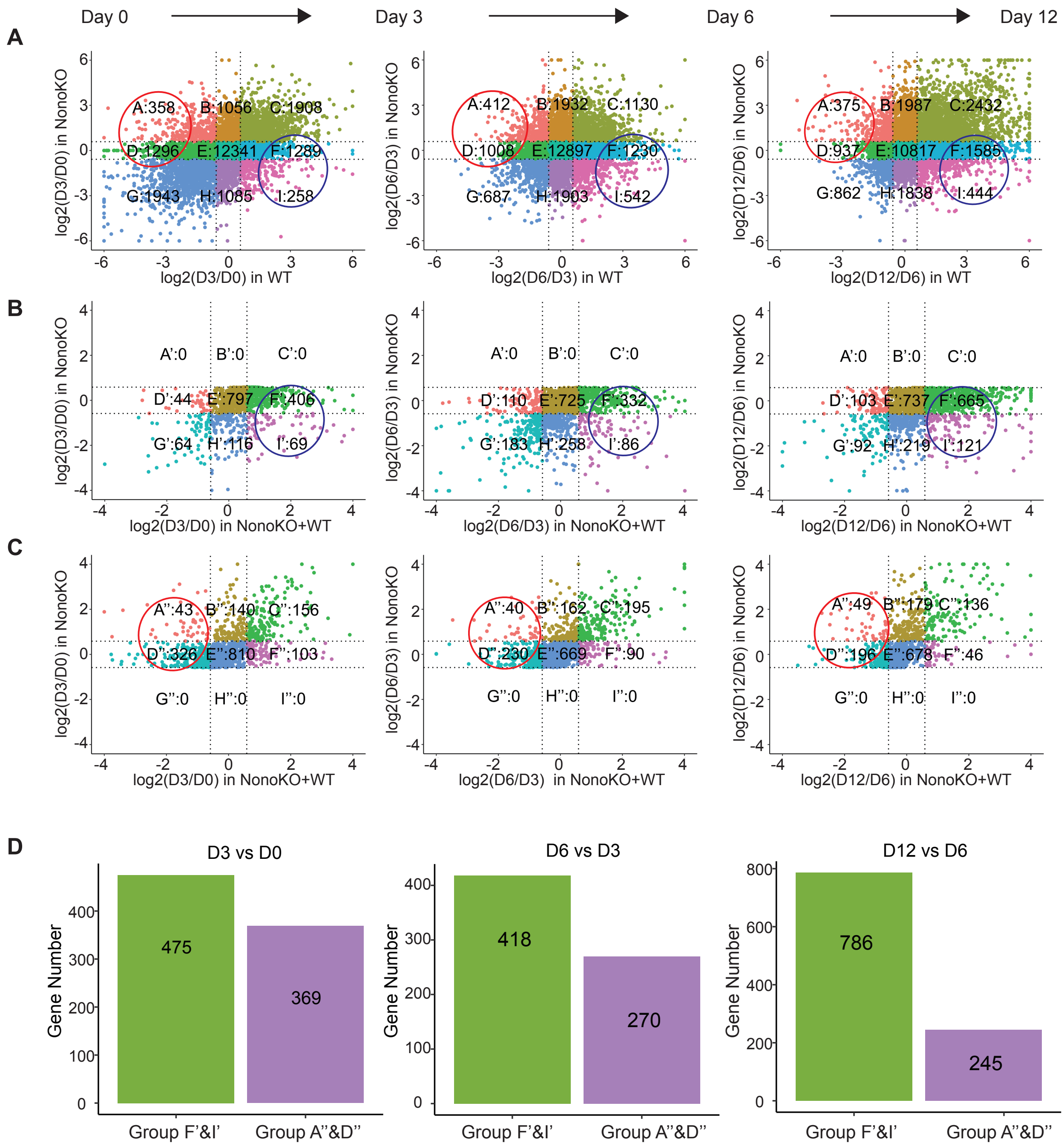

Supplementary Figure 3

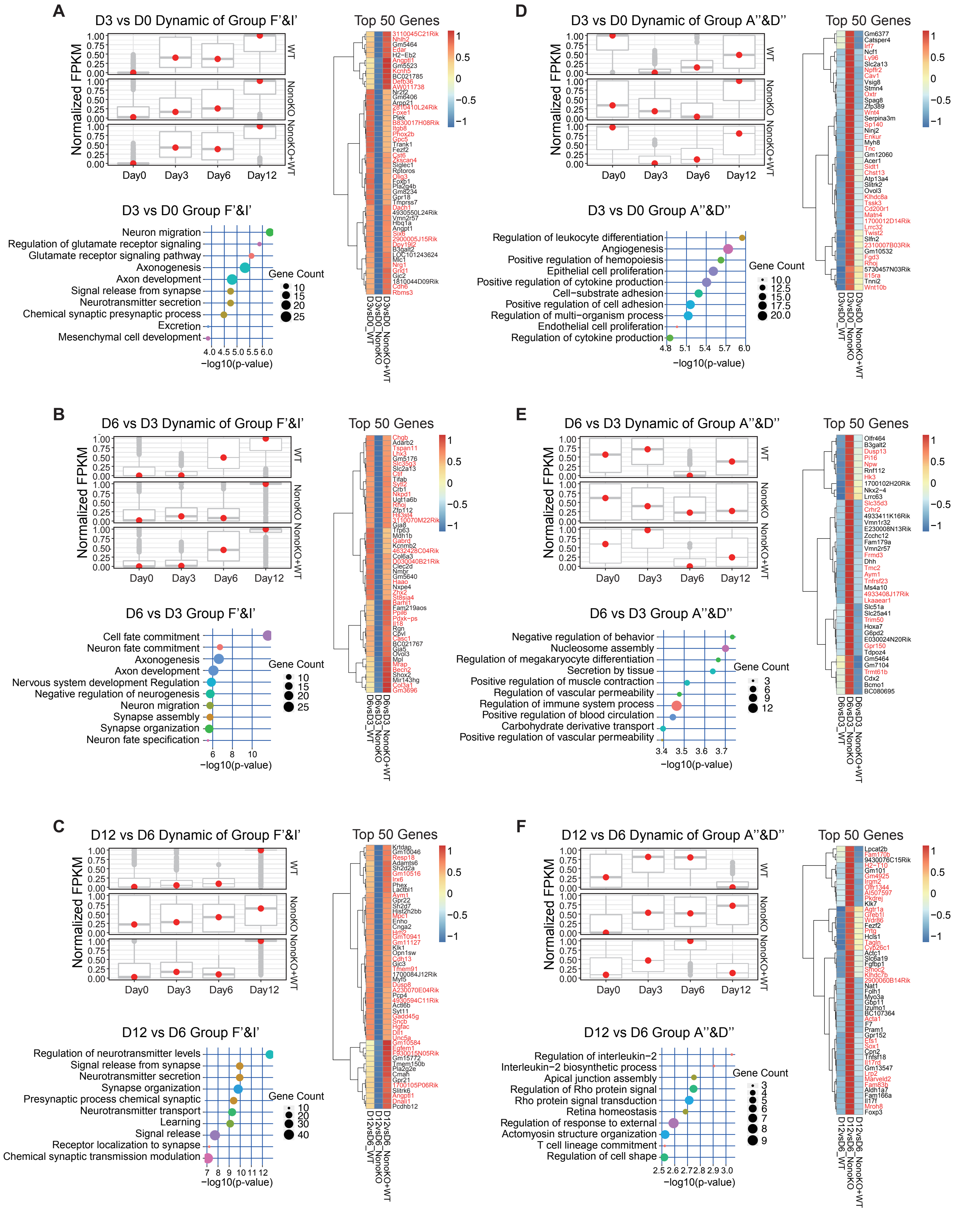

Supplementary Figure 4

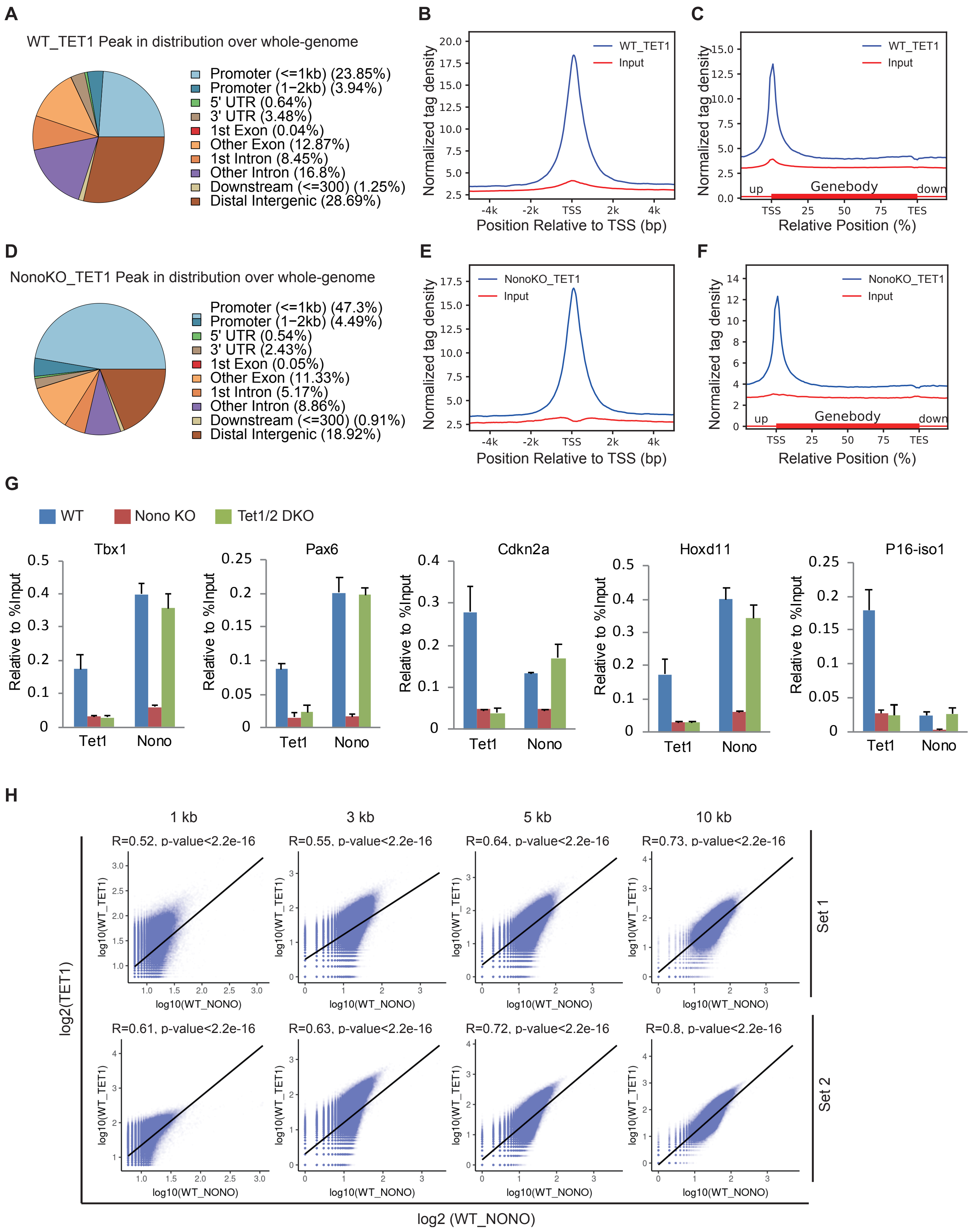

Supplementary Figure 5

A

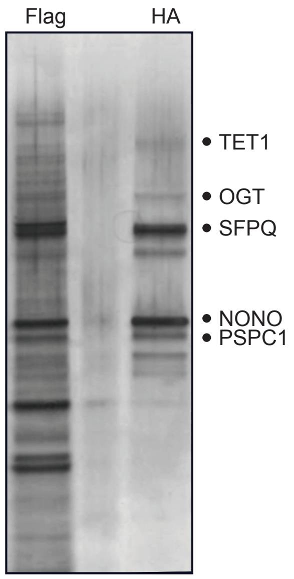

B

	Unique Peptide Number	
Protein name	Flag-HA mES	Endogeous mES
NONO	125	11
SFPQ	103	13
PSPC1	15	2
HNRNPU	12	13
D1PAS1	10	5
HNRNPA3	9	3
OGT	6	2
TET1	3	1
FUS	4	2

C

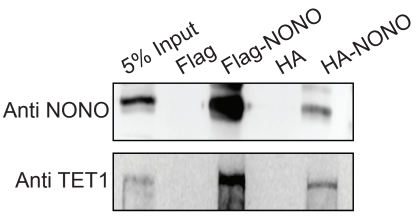

Supplementary Figure 6

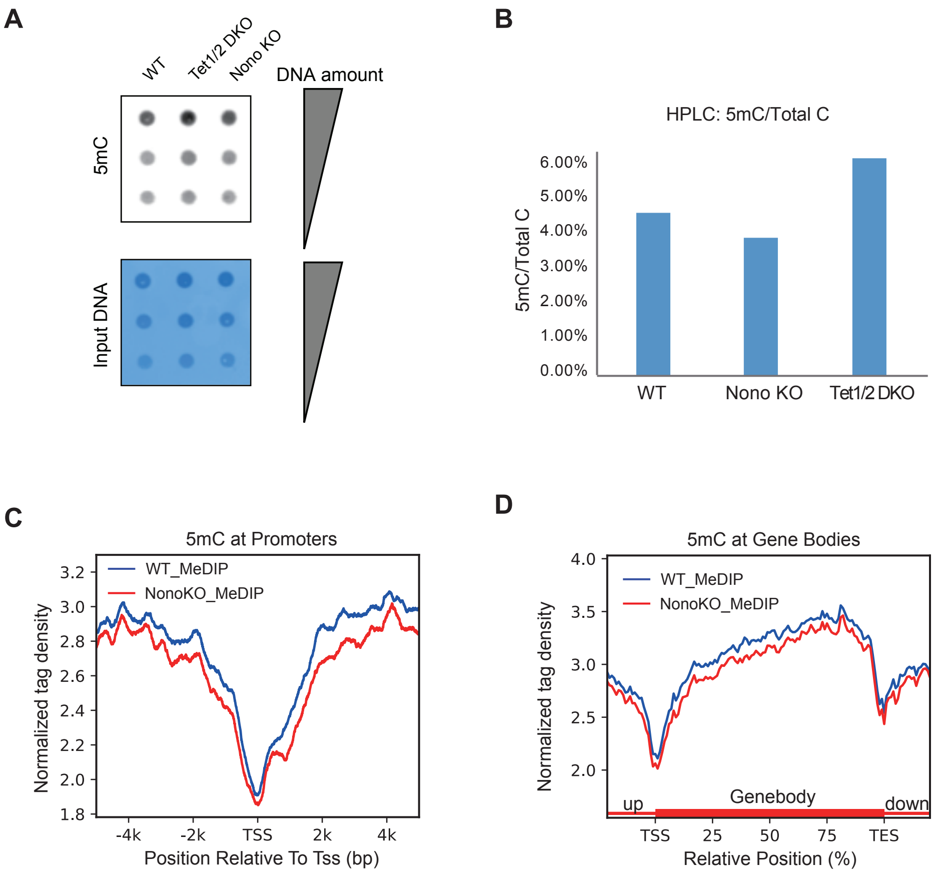

Supplementary Figure 7

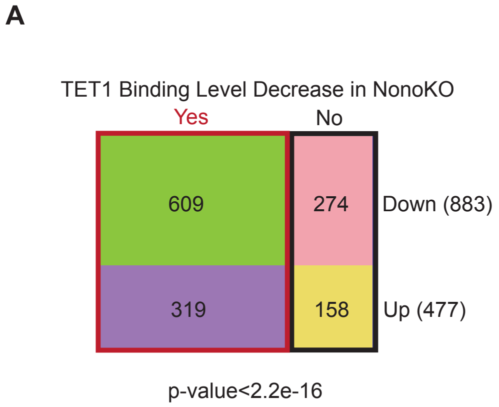

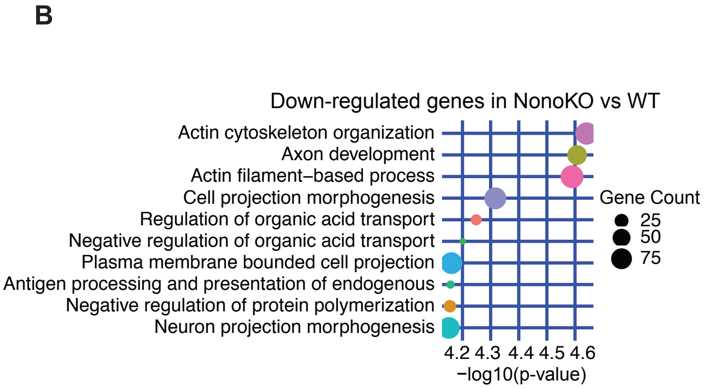

Supplement: gkaa213_Supplemental_File [file gkaa213_supplemental_file.pdf]
